# Discovery and Preclinical Activity of BMS-986351, an Antibody to SIRPα That Enhances Macrophage-mediated Tumor Phagocytosis When Combined with Opsonizing Antibodies

**DOI:** 10.1158/2767-9764.CRC-23-0634

**Published:** 2024-02-22

**Authors:** Henry Chan, Christina V. Trout, David Mikolon, Preston Adams, Roberto Guzman, Konstantinos Mavrommatis, Mahan Abbasian, Haralambos Hadjivassiliou, Lawrence Dearth, Brian A. Fox, Pallavur Sivakumar, Ho Cho, Kandasamy Hariharan

**Affiliations:** 1 Discovery Biotherapeutics, Bristol Myers Squibb, San Diego, California.; 2 Strategy and Business Development, Avidity Biosciences, Inc., San Diego, California.; 3 Takeda, San Diego, California.; 4 Computational Genomics, Bristol Myers Squibb, San Francisco, California.; 5 Yatiri Bio, San Diego, California.; 6 Informatics and Predictive Sciences, Bristol Myers Squibb, Seattle, Washington.; 7 Immuno-Oncology and Cell Therapy Discovery, Bristol Myers Squibb, Seattle, Washington.; 8 Samsung Bioepis, Seoul, Republic of South Korea.

## Abstract

In normal cells, binding of the transmembrane protein CD47 to signal regulatory protein-α (SIRPα) on macrophages induces an antiphagocytic signal. Tumor cells hijack this pathway and overexpress CD47 to evade immune destruction. Macrophage antitumor activity can be restored by simultaneously blocking the CD47-SIRPα signaling axis and inducing a prophagocytic signal via tumor-opsonizing antibodies. We identified a novel, fully human mAb (BMS-986351) that binds SIRPα with high affinity. BMS-986351 demonstrated broad binding coverage across SIRPα polymorphisms and potently blocked CD47-SIRPα binding at the CD47 binding site in a dose-dependent manner. *In vitro*, BMS-986351 increased phagocytic activity against cell lines from solid tumors and hematologic malignancies, and this effect was markedly enhanced when BMS-986351 was combined with the opsonizing antibodies cetuximab and rituximab. A phase I dose-escalation/-expansion study of BMS-986351 for the treatment of advanced solid and hematologic malignancies is underway (NCT03783403).

**Significance:**

Increasing the phagocytotic capabilities of tumor-associated macrophages by modulating macrophage–tumor cell surface signaling via the CD47-SIRPα axis is a novel strategy. Molecules targeting CD47 have potential but its ubiquitous expression necessitates higher therapeutic doses to overcome potential antigen sink effects. The restricted expression pattern of SIRPα may limit toxicities and lower doses of the SIRPα antibody BMS-986351 may overcome target mediated drug disposition while maintaining the desired pharmacology.

## Introduction

Macrophages are innate immune cells that play a key role in homeostasis and the elimination of foreign, aged, or damaged cells through phagocytosis (1). To prevent macrophages from attacking healthy tissue, normal cells express cell-surface markers that inhibit phagocytic activity. One such cellular marker, CD47, is a ubiquitously expressed membrane protein often referred to as the “don't eat me” molecule (2, 3). CD47 blocks phagocytosis by binding to signal regulatory protein-α (SIRPα) found on the surface of macrophages (2, 4). Upon binding to CD47, SIRPα tyrosine-based inhibition motifs recruit src-homology-2 domain-containing tyrosine phosphatases 1 and 2 (SHP1 and SHP2), which cleave phosphate groups from the tyrosine-based activation motifs of Fc receptors on macrophages (5–7). This process prevents phagocytosis via the dephosphorylation of myosin-II in macrophages (8). Thus, the binding of CD47 and SIRPα represents an important immune checkpoint needed to maintain immune homeostasis (2, 9, 10).

Cancer cells upregulate CD47 expression on their surface to avoid undergoing phagocytosis as a form of immune evasion (3, 9, 11, 12). Clinically, CD47 overexpression has been associated with worse prognoses in some cancer types (13–15), further supporting the role of this pathway in tumorigenesis. Targeting the CD47-SIRPα axis could therefore restore immune function and promote antitumor effects (16, 17). Numerous strategies that target CD47 in solid and hematologic malignancies are in development (17–26), and preclinical evidence suggests that disrupting CD47 signaling promotes phagocytosis, inhibits tumor growth and survival, and enhances the antitumor activity of opsonizing therapeutic antibodies (9, 17). However, the therapeutic window of agents that target CD47 is limited because CD47 is ubiquitously expressed. High doses are needed to overcome the antigen sink, and these, coupled with target expression on platelets, red blood cells (RBC), and other healthy cells, may lead to undesirable adverse events (10, 11). In addition, CD47 is known to directly interact with multiple factors besides SIRPα, including other extracellular ligands [SIRPγ, thrombospondin-1 (TSP1), serpin A1], membrane-associated integrins, and intracellular factors (Syk, ERK, PI3K; ref. 16), potentially resulting in toxicities.

An alternative strategy is to target SIRPα, which has a much more restricted pattern of expression than CD47 and could potentially reduce the risk of on-target, off-tumor toxicities (10, 12). SIRPα has two homologs: SIRPβ and SIRPγ. Like SIRPα, SIRPβ is expressed on monocytes, but it does not bind to CD47, and its function is not well understood (16). SIRPγ is expressed primarily on T cells and binds to CD47 (with a lower affinity than SIRPα) to promote cell-cell adhesion and T-cell migration and activity (10, 16, 27–29).

The inhibition of antiphagocytic signaling through the CD47-SIRPα blockade allows macrophages to exert their antitumor effects. In addition, the blocking of the antiphagocytic signaling may be enhanced by concomitant use of opsonizing therapeutic antibodies (such as cetuximab or rituximab), which promote prophagocytic signaling by providing a secondary activating signal to induce phagocytosis via macrophage Fcγ receptors (FcγR; refs. 17, 30, 31).

In this article, we identify BMS-986351, a novel, high-affinity, fully human mAb with modified effector function targeting SIRPα. We also characterize its effect on phagocytic activity, alone or in combination with opsonizing antibodies, in several solid tumor and hematologic malignancy models.

## Materials and Methods

### Modeling Interaction of CD47 with SIRPα, and BMS-986351-Fab with SIRPα

**FIGURE 1:**
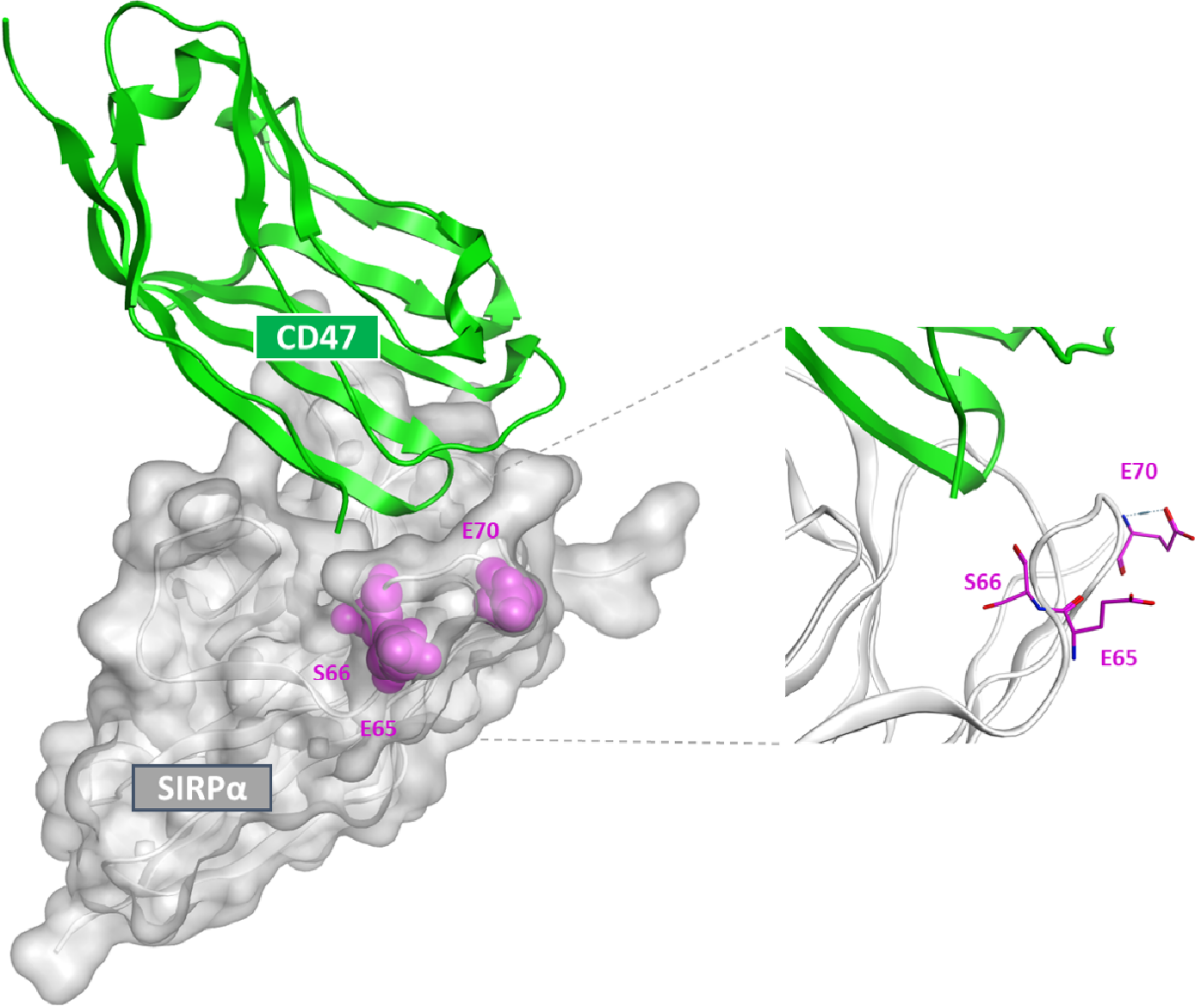
Modeling of interactions between the CD47 F-G loop and the SIRPα IgV domain, with the position of the ESE haplotype residues highlighted (magenta).

**FIGURE 2:**
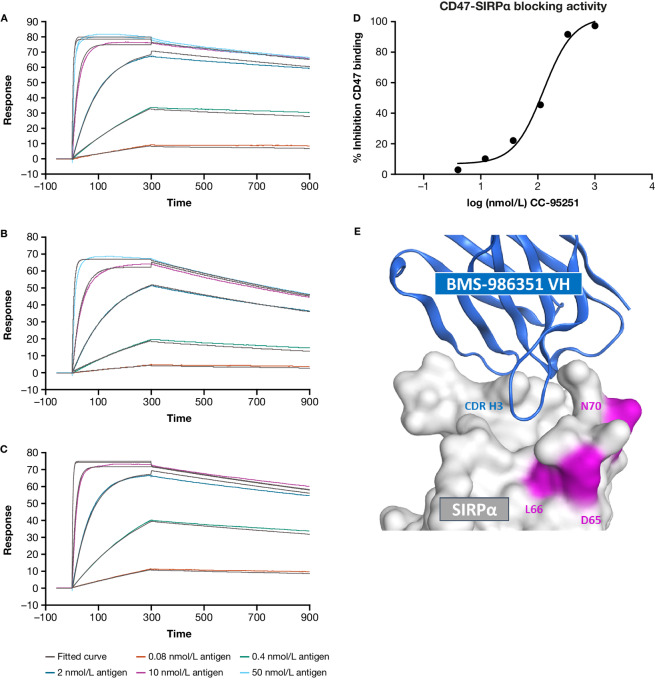
Binding affinity of BMS-986351 to SIRPα (**A**), SIRPβ (**B**), and SIRPγ (**C**) as determined by SPR and inhibition of CD47-SIRPα binding (**D**). **E,** Cocrystallization of BMS-986351 with SIRPα (structure available in the Protein Data Bank, entry 7ST5) indicates that the site of interaction between the Fab epitope (the CDR H3 loop) of BMS-986351 (blue) and the SIRPα IgV domain overlaps considerably with the CD47-SIRPα binding domain. The positions of the DLN motif haplotype residues, away from the site of interaction, are highlighted in magenta.

Models of the interaction between the CD47 F-G loop and the SIRPα immunoglobulin (Ig) V domain (ESE haplotype, PDB 2JJS; Fig. 1) and between BMS-986351-Fab and the SIRPα*-*extracellular domain 1 (ECD1; DLN haplotype, PDB 7ST5; Fig. 2) were generated using Molecular Operating Environment (Chemical Computing Group).

### Haplotype Identification and Assessment of Abundance

The haplotype analysis and subsequent results were focused on the subregion of the variable region (commonly referred as the DLN region), which has been shown to interact with CD47. To identify the SIRPα haplotypes and their abundance, genomic sequences from 2504 individuals from the 1000 Genome (1kG) project phase III were used. Genomic sequences were analyzed against the internal BMS pipeline, including alignment to the human genome (h19), using BWA v0.7.9 (32), and detection of germline mutations, using Samtools v0.19 (32), and filtered at a Phred score of 30. The detected mutations were phased using the GATK ReadBackedPhasing (v3.2) tool (33) and annotated using SNPEFF v3.1 (34). Haplotypes that contained frameshift mutations resulting in truncated forms of the protein were excluded. Of the remaining sequences, clustering at 100% identity produced 92 representative haplotypes. Clustering was performed using USEARCH (35). The most abundant combinations of representative haplotypes are presented in Supplementary Table S1.

### Identification and Activity of Lead Anti-SIRPα Antibody (BMS-986351)

Screening and discovery of fully human IgG antibodies against SIRPα were performed at Adimab, LLC, using yeast display platform selections. The extracellular domain of recombinant human SIRPα was used as bait to isolate binders from eight libraries totaling approximately 10^10^ fully human IgG antibodies expressed on yeast cells. Heavy chains from selection outputs were further shuffled against nine light-chain libraries for additional diversity. Successive rounds of selective sorting yielded >1,300 naïve isolates for sequencing, and 564 were identified for production, binding, and CD47-blocking analysis. Internal testing identified seven IgG binders for affinity maturation; focused complementarity-determining region heavy chain 1 and 2 libraries for each lineage (each >10^8^) were paired with their corresponding light chains and screened for high-affinity human and cynomolgus monkey SIRPα binders. More than 350 “offspring” from six parental lineages were sequenced and produced for characterization of SIRPα binding, polymorphic coverage, and cross-reactivity profiles. The top 24 clones across six lineages underwent characterization, including cell binding, affinity determination by surface plasmon resonance (SPR), ligand blocking, and *in vitro* functional assays, and lead and backup antibodies were selected. The sequence of BMS-986351 is described in the Protein Data Bank, PDB 7ST5 (36), and patent US 11,591,390 B2 (37); experimental details are briefly summarized in the Supplementary Materials and Methods.

### Binding Affinity Analysis (SPR): BMS-986351 and SIRPα, SIRPβ, and SIRPγ

Direct affinity measurements of BMS-986351 binding to recombinant extracellular domains of SIRPα, SIRPβ, and SIRPγ were performed by SPR on a Biacore T200. Purified BMS-986351 was captured onto a ProA sensor chip on channel 2, and recombinant SIRPα, SIRPβ, or SIRPγ proteins were injected into both channels 1 and 2 in 5-fold dilutions (0.08–50 nmol/L) to measure the resonance signal over 900 seconds. The surface was regenerated between each antigen concentration using 10 mmol/L glycine, pH 1.5. Kinetic binding data were analyzed using Biacore BIAevaluation software with a 1:1 Langmuir model.

### BMS-986351 CD47-blocking Activity

The ability of BMS-986351 to block the binding of SIRPα to CD47 was analyzed using SPR. Human recombinant CD47-ECD was immobilized on ProA sensors, and samples of premixed human recombinant SIRPα (20 nmol/L) with serially diluted BMS-986351 were flowed over channels 1 and 2. The resulting binding data were analyzed with the Affinity-in-Solution model using the Biacore T200 BIAevaluation software. On the basis of the measured free concentration of SIRPα able to bind immobilized CD47-ECD, the calculated Bound concentration equilibrium (Beq), % inhibition, and IC_50_ values were calculated.

### Binding Coverage of BMS-986351 Across Haplotypes

Binding of papain-derived and purified Fab of BMS-986351 to captured SIRPα variants 1–6 Fc was measured by Octet BLI under affinity conditions. SIRPα variants expressed as Fc fusion proteins were captured on anti-human capture tips and dipped into 50 nmol/L BMS-986351-Fab; association was measured for 180 seconds; dissociation was measured in buffer for an additional 180 seconds; 0.0002 (2 × 10^−4^) is the maximum off-rate (k_off_) that can be confidently assigned under the assay conditions.

### Expression, Purification, Crystallization, and Structure Determination of SIRPα ECD1 and BMS-986351-Fab

The gene encoding for ECD1 of human SIRPα (residues 31–149, UNIPROT identifier P78324-1, isoform 1) was synthesized by GENSCRIPT and codon-optimized for expression in *S. frugiperda* (Sf9) cells. The *SIRPα* gene construct included an N-terminal signal sequence peptide from glycoprotein gp67 engineered to secrete protein into the media during expression, a thrombin cleavage site, and a 6x histidine tag at the C-terminus to facilitate purification. Recombinant baculoviruses were generated using the Bac-to-Bac system (Invitrogen) and used to infect Sf9 cells. Infected cells were grown at 27°C for 48 hours in the presence of 10 µmol/L kifunensine. SIRPα-ECD-D1 was purified from culture supernatants using a 5 mL Ni-NTA column (cOmplete His-Tag purification resin, Roche) pre-equilibrated with a buffer containing 25 mmol/L HEPES pH 7.0 and 150 mmol/L NaCl in an Äcta Pure chromatography system. Elution was performed using 25 mmol/L HEPES pH 7.0 and 150 mmol/L NaCl with an imidazole gradient (0.025–1 mol/L). Fractions containing the protein of interest were pooled and deglycosylated overnight at 4°C using endoglycosidase H enzyme (New England BioLabs), and subsequently dialyzed in a buffer containing 25 mmol/L HEPES pH 7.0 and 10 mmol/L NaCl. The sample was further purified using a MonoS 10/100 ion exchange chromatography column (GE Healthcare) with 25 mmol/L HEPES pH 7.0 and an NaCl gradient (0.025–1 mol/L) for elution. Fractions containing purified SIRPα ECD1 were pooled and immediately used in complex formation experiments. The Fab fragment from the antibody BMS-986351 was prepared using the Fab Preparation Kit (Pierce) and mixed at equal stoichiometric ratios with purified SIRPα ECD1 before a final size exclusion chromatography step on a Superdex 200 16/600 column using 10 mmol/L HEPES pH 7.5 and 25 mmol/L NaCl.

For crystallization experiments, the SIRPα-ECD1 DLN/BMS-986351–Fab complex was concentrated to 15 mg/mL. Crystallization screens were performed using the hanging drop method in a 96-well plate format. Crystals were obtained at 20°C using the vapor diffusion sitting drop crystallization method and a precipitant solution containing 0.1 mol/L MES pH 6.5, 25% w/v PEG MME 550, and 0.01 mol/L zinc sulfate. Crystals were cryoprotected using 20% ethylene glycol and flash-cooled under liquid nitrogen. Diffraction data from crystals were collected at the Argonne National Laboratory Advanced Photon Source, APS LS-CAT 21-ID-D.

Diffraction data were processed using the XDS Program Package and refined using the REFMAC5 software as part of the CCP4 suite. The crystal structure of the SIRPα Fab complex was determined by molecular replacement using the PHASER software.

### SIRPα Prevalence in Tumor Samples

Gene expression data from The Cancer Genome Atlas (TCGA; ref. 38) were downloaded from UCSC Xena Toil (39). The gene-level normalized count data were transformed by adding a pseudocount of 1 to all values and then applying a log_2_ transformation. The genes and indications of interest were extracted, and scatterplots were made from those data.

Tissue microarrays used for IHC analysis were MTU 951, LYM 1022, COC 1021, and HNT1021 (Pantomics). Formalin-fixed paraffin-embedded tissues were sectioned at 4-µm-thick and deparaffinized on the Bond-III instrument (Leica Biosystems). Antigen retrieval was performed with Epitope Retrieval Solution 2 pH 9.0 at 100°C for 20 minutes. Slides were blocked for endogenous peroxidase activity before sections were incubated with anti-SIRPα (rabbit polyclonal 1/1600; Abcam) or anti-CD163 (mouse monoclonal 1/200; Novus Biologicals) primary antibodies for 15 minutes. Both of these commercial antibodies have been previously validated against normal tissue panels, showing that staining was limited to resident phagocytic cells in multiple tissues. The post-primary rabbit anti-mouse IgG and anti-rabbit horseradish peroxidase (HRP)-polymer labeled IgG antibodies were incubated, using the instrument's default conditions. The antigen–antibody complex was then visualized with hydrogen peroxide substrate and diaminobenzidine tetrahydrochloride (DAB) chromogen before being counterstained with hemaoxylin, dehydrated, and coverslipped by the Tissue-Tek Film Automated Coverslipper (Sakura Finetek).

### *In Vitro* Phagocytic Activity of BMS-986351

Negatively selected human monocytes (Astarte Biologics) were cultured and differentiated into macrophages with 50 ng/mL MCSF containing AIM-V medium for 6–9 days. The effect of BMS-986351 on promoting phagocytosis of tumor cells by human macrophages in combination with either cetuximab or rituximab was assessed *in vitro* by coculture assays. A total of 40,000 tumor cells labeled with carboxyfluorescein diacetate succinimidyl ester (CFSE) were opsonized with cetuximab or rituximab and added to each well containing 40,000 human macrophages preincubated with BMS-986351. BMS-986351 was preincubated for 10 minutes at room temperature at a concentration of 20 nmol/L with additional concentrations at 3- or 10-fold dilutions. BMS-986351 remained present for the 3-hour duration of the phagocytosis assay, which included the macrophages and tumor cells in coculture. After 3 hours, the macrophages with tumor cells were washed, and the surface was labeled with anti-CD14-allophycocyanin (APC). Subsequent tumor phagocytosis was captured using an Operetta High Content Imager, detecting CFSE in the fluorescein isothiocyanate channel and APC in the Alexa Fluor 647 channel. Operetta Harmony analysis software was used to calculate the percentage of double-positive phagocytotic macrophages. In this manner, four colorectal cancer cell lines and one squamous cell carcinoma of the head and neck (SCCHN) line (ATCC; obtained in 2016 or 2017 and tested for *Mycoplasma* bimonthly prior to liquid N_2_ storage) were assayed for the combined effect of BMS-986351 with cetuximab (Eli Lilly), and four diffuse large B-cell lymphoma (DLBCL) lines were assayed for the combination effect with rituximab (Genentech/Roche). For testing of antibody-dependent cellular phagocytosis (ADCP) of monocytes or T cells with BMS-986351, 40,000 donor-matched monocytes or T cells were stained with CFSE and incubated with 40,000 macrophages in the same manner as for the tumor cell coculture studies, using the same analysis methods.

### FcγR Binding Assessment

BMS-986351 binding to different FcγRs expressed on HEK293 cells was measured using a time-resolved fluorescence resonance energy transfer (FRET) assay (Cisbio).

IgG-d2-acceptor conjugate binding to the Tb donor cells generates a specific FRET signal, which may be displaced by test articles. The specific signals (665 nm/620 nm) were detected with an Envision 2104 multilabel reader to determine the FRET signals, and binding EC_50_ values were calculated following the manufacturer's protocol.

### Species Cross-reactivity

#### Direct Staining

Binding of BMS-986351 to human and cynomolgus SIRPα-overexpressing CHO-K1 cells was assessed by incubating with directly conjugated BMS-986351-AF647 (Thermo Fisher Scientific). Geometric mean fluorescent intensity (gMFI) on the APC channel was quantified by flow cytometry for each duplicate sample and plotted against antibody concentration.

#### Indirect Staining

Binding of BMS-986351 to rat and mouse SIRPα-overexpressing CHO-K1 cells were assessed by detection with secondary anti-human AF647 secondary antibodies (Jackson ImmunoResearch). gMFI on the APC channel was plotted against antibody concentration. Clone OX-41 (BioLegend) anti‑rat SIRPα was used to detect rat SIRPα-overexpressing CHO-K1 cells, and clone P84 (BioLegend) was used as a positive control for to detect mouse SIRPα.

### Immunophenotyping by Multiparameter Flow Cytometry

BMS-986351 binding to peripheral blood mononuclear cells (PBMC) from five humans and five cynomolgus macaques was assessed by multiparameter flow cytometry. Human/cynomolgus macaque surface cross-reactive antibodies were selected for the flow cytometry panel, with the same gating scheme applied to both species. The gMFIs of BMS-986351 and an isotype-matched control within each immune subset were averaged and plotted.

### Antibody-dependent Cell-mediated Cytotoxicity Activity Assay

Donor human PBMCs were isolated from healthy volunteers (Scripps Research Institute blood donor service) using Ficoll-Paque (GE Healthcare Life Sciences) and preactivated overnight with recombinant hIL-2 (R&D Systems). The CD33^+^ SIRPα^+^ MOLM-13 (DSMZ, Braunschweig, Germany) tumor cell line (Target) was prelabeled with CellTrace Violet (Thermo Fisher Scientific) and preincubated with test antibodies at dose concentrations up to 200 nmol/L before coculturing with PBMCs at a final effector-to-target ratio of 80:1 for 3 hours at 37°C. Propidium iodide (PI) was added to the coculture, the target cytotoxicity was determined using a Mirroball cytometer, and data were analyzed using Cellista V4.33 (SPT Labtech) as CellTrace Violet/PI-positive versus total target cells.

### BMS-986351 Pharmacokinetics and Hematologic Effects *In Vivo*

A sandwich ELISA immunoassay was developed to measure the concentration of BMS-986351. The assay uses recombinant SIRPα fusion protein for capture and HRP-conjugated donkey anti-human IgG H+L antibody for detection. The lower limit of quantitation for this method is 100 ng/mL.

Toxicokinetic parameters were calculated using Watson LIMS 7.5 (Thermo Fisher Scientific). The peak serum concentration (C_max_), time at peak serum concentration (T_max_), total body clearance (CL), volume of distribution (Vdss), and half-life (T_½_) were determined for each animal after each dose. The area under the serum concentration–time curve from 0 to 168 hours (AUC_0–168_) was determined by the noncompartmental model using the linear/log trapezoidal rule for day 1 and day 15, respectively. Serum concentrations below the limit of quantitation were not used for AUC calculations.

Hematology parameters, such as white blood cell (WBC), monocyte, lymphocyte, and RBC counts, were measured periodically over 28 days after exposure to BMS-986351 in cynomolgus monkeys. All cynomolgus monkey studies described were approved by an Institutional Animal Care and Use Committee review board.

### Data Availability

The data from TCGA and the 1000 Genome dataset that were analyzed in this study were obtained from https://xenabrowser.net/datapages/?dataset=tcga_target_no_normal_RSEM_hugo_norm_count&host=https%3A%2F%2Ftoil.xenahubs.net and EMBL European Bioinformatics Institute https://www.ebi.ac.uk/, respectively. The raw data underlying the included SPR results were generated by a core facility and are no longer available. Other data are available upon reasonable request to the lead author, Henry Chan (henry.chan@bms.com) and review by BMS, in compliance with BMS compound, technology, and data sharing policies (further details can be found at https://www.bms.com/researchers-and-partners/independent-research/compound-and-technology-requests.html and https://www.bms.com/researchers-and-partners/independent-research/data-sharing-request-process.html).

## Results

### Target Characterization and Haplotype Analysis

The crystal structure of the CD47-SIRPα interface has been reported previously (40). To understand the structural determinants within the SIRPα IgV binding loop (amino acids 76–106) through the impact of the six most highly represented haplotypes (representing >95% of the human population; Supplementary Table S1), we performed molecular docking for each of the six haplotypes in relation to the CD47 F-G loop (Fig. 1, SIRPαv2 ESE haplotype). On the basis of their position away from the CD47 F-G loop, we determined that the DLN motif, and the corresponding haplotypes, are unlikely to impact on CD47-SIRPα binding.

### Identification and Activity of Lead Anti-SIRPα Antibody (BMS-986351)

Initial screening and discovery of fully human IgG antibodies against SIRPα were performed using a yeast display platform. Selective sorting yielded more than 1,300 naïve isolates for sequencing, which were further filtered to about 350 candidates from six parental lineages. The top 24 clones were fully characterized, and a lead mAb (BMS-986351) emerged that exhibited binding to SIRPα, SIRPβ, and SIRPγ, with K_D_ values of 0.0541, 0.205, and 0.0502 nmol/L, respectively (Fig. 2A–C; Supplementary Table S2). BMS-986351 contains a human IgG1 Fc with a lysine to alanine amino acid substitution at position 322 (K322A) that suppresses C1q binding (41) and reduces its ability to activate complement while maintaining all other effector functions.

BMS-986351 blocked CD47-SIRPα binding *in vitro* in a dose-dependent manner, with a concentration of 100 nmol/L almost completely inhibiting CD47 binding activity (Fig. 2D; Supplementary Table S3). BMS-986351 also demonstrated binding to human FcγR1–3, with EC_50_ values comparable to wild-type IgG1-containing anti-respiratory syncytial virus (RSV) and rituximab antibody controls (Supplementary Table S4).

BMS-986351 exhibited strong binding coverage across the six most prevalent SIRPα haplotypes (Supplementary Table S5) identified from analysis of 1kG project data. Cocrystallization modeling of BMS-986351 and SIRPα revealed that the Fab epitope of BMS-986351 interacts with SIRPα in a region that substantially overlaps with the binding site for CD47, demonstrating a mechanism by which BMS-986351 blocks SIRPα–CD47 interactions (Fig. 2E).

### SIRPα Prevalence in Tumor Samples

**FIGURE 3:**
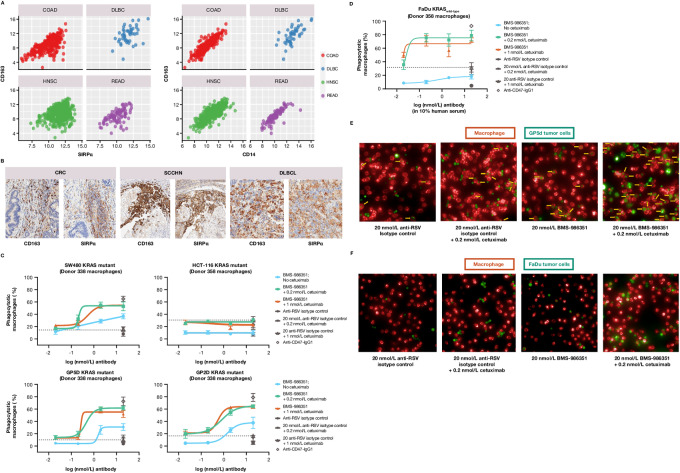
Gene expression data from TCGA across four indications (COAD = colon adenocarcinoma, DLBC = diffuse large B-cell lymphoma, HNSC = head and neck cancer, READ = rectal adenocarcinoma) with each point from one patient and expression units shown as the log_2_(normalized counts+1). The left panel of (**A**) shows that the absolute level of SIRPα (*x*-axis) is comparable to and often correlated with CD163 gene expression (*y*-axis). The right panel of (**A**) shows that CD163 is a representative marker of myeloid cells as it is highly correlated to CD14 (*x*-axis), with many other macrophage marker genes showing a similar pattern (not shown). Validation by IHC in tumor samples from CRC, SCCHN, and DLBCL (**B**), showing overlapping expression of macrophage marker CD163 and SIRPα (data shown are example images from the tumor microarray). Proportion of phagocytic macrophages after exposure to BMS-986351 alone or in combination with cetuximab in samples derived from donors with CRC (**C**) and SCCHN (**D**). Immunofluorescence illustrating increased tumor cell phagocytosis with BMS-986351 plus cetuximab compared with cetuximab plus isotype matched control (anti-RSV) or control alone in GP5d (**E**) and FaDu (**F**) tumor cells. Arrows indicate phagocytic macrophages. CRC, colorectal cancer; IHC, immunohistochemistry; RSV, respiratory syncytial virus; SCCHN, squamous cell carcinoma of head and neck.

Anti-CD47 efficacy can be potentiated by the activation of a prophagocytic signal from opsonizing antibodies such as cetuximab and rituximab (22, 42). Therefore, this study focused on tumors that already had clinical approval for the use of opsonizing antibodies, expressed CD47-SIRPα, and demonstrated macrophage infiltration. Three indications were prioritized because of clinical approval of opsonizing antibodies: colorectal cancer and SCCHN for cetuximab and DLBCL for rituximab. To determine whether these tumor types may be susceptible to disruption of the CD47-SIRPα axis using this strategy, gene expression levels of CD47-SIRPα and CD163 (a marker of monocyte/macrophage infiltration) were assessed in bulk tumor samples using data from TCGA. The absolute gene expression level of the *SIRPα* gene was comparable to that of *CD163* and was correlated with that of *CD163* in the prioritized indications (Fig. 3A), which suggested that *SIRPα* expression would be higher in tumors with higher macrophage infiltration. We and others have also found that *CD163* can be used to generally represent myeloid or macrophage expression because it is highly correlated to *CD14* and many other macrophage genes (Fig. 3A; ref. 43). SIRPα expression was also correlated to that of other macrophage genes in TCGA samples for the prioritized indications (Supplementary Table S6). *CD47* gene expression was found to be consistently high across all tumor indications profiled in TCGA, with a narrow range of variability (Supplementary Fig. S1A). The analysis identified that the prioritized tumor types, that is, colorectal cancer, SCCHN, and DLBCL, had tumors with higher levels of CD47-SIRPα and macrophage infiltration, and thus may be suitable targets for BMS-986351 treatment. In addition, other tumor indications, such as renal cell carcinoma, glioblastoma, metastatic melanoma, and mesothelioma, displayed high expression of both *SIRPα* and macrophage genes (Supplementary Fig. S1B).

Protein levels of SIRPα and CD163 were additionally confirmed by IHC in tumor samples from patients with colorectal cancer, SCCHN, and DLBCL (Fig. 3B). Tumor microarray samples were positive for tumor-associated macrophages (CD163) and neutrophils (CD66b) in colorectal cancer (*n* = 97), SCCHN (*n* = 80), and DLBCL (*n* = 75; Supplementary Table S7A). For SCCHN, weak to strong staining for both SIRPα (83%) and EGFR (90%) was detected in the neoplastic cells (Supplementary Table S7B). SIRPα and CD163 expression were also evaluated by IHC in a panel of 75 DLBCL samples; SIRPα was observed in 28%, with most of the positive samples having an H score of 100–200 (Supplementary Table S7C).

### BMS-S986351 Treatment Alone and in Combination with Cetuximab in Colorectal Cancer and SCCHN

The impact of BMS-986351 on macrophage phagocytic activity was assessed *in vitro* using differentiated macrophages cocultured with various cetuximab-resistant tumor cell lines, including four colorectal cancer cell lines [GP2d, GP5d (KRAS^G12D^), SW480 (KRAS^G12V^), and HCT116 (KRAS^G13D^)] and one SCCHN cell line [FaDu (KRAS^wild-type^)]. Baseline CD47 expression was robust in the four colorectal cancer cell lines tested (Supplementary Fig. S2A). Exposure to BMS-986351 alone increased the proportion of phagocytic macrophages in a dose-dependent manner in three out of four of the colorectal cancer cell lines compared with an anti-RSV control (Fig. 3C). A limited increase in phagocytic macrophages was seen with HCT116 cells, suggesting that these cells may interact with macrophages via SIRPα-independent mechanisms. In FaDu cells, derived from hypopharyngeal carcinoma, BMS-986351 led to a modest increase in phagocytic macrophages when used as monotherapy, and a marked increase when given in combination with cetuximab (Fig. 3D). Immunofluorescence analyses illustrated the increased phagocytic activity observed when GP5d cells (Fig. 3E) or FaDu cells (Fig. 3F) cocultured with macrophages were exposed to both BMS-986351 and cetuximab.

### BMS-986351 Treatment Alone and in Combination with Rituximab in DLBCL

**FIGURE 4:**
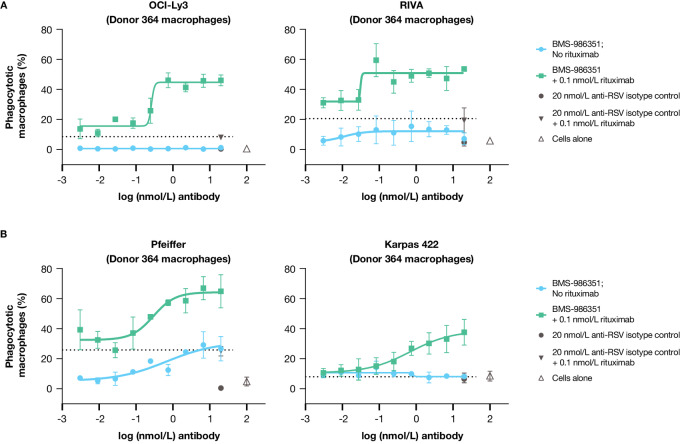
Effect of BMS-986351, alone or in combination with rituximab, on the proportion of phagocytic macrophages in ABC (**A**) and GCB (**B**) cell lines. ABC, activated B cell; GCB, germinal center B cell.

The impact of BMS-986351 alone and in combination with rituximab on the proportion of phagocytic macrophages was assessed in four CD20-positive DLBCL cell lines cocultured with differentiated macrophages. The concentration of 0.1 nmol/L rituximab used in these experiments was determined via cell culture titration assays measuring levels of macrophage phagocytosis at varying rituximab concentrations (Supplementary Fig. S3). The cell lines included two activated B-cell (ABC) subtypes (OCI-Ly3 and RIVA; Fig. 4A) and two germinal center B-cell (GCB) subtypes (Pfeiffer and Karpas 422; Fig. 4B). Baseline CD47 expression was robust in the four DLBCL cell lines tested (Supplementary Fig. S2B). Exposure to BMS-986351 or rituximab alone had varying but overall limited effects on the levels of phagocytic macrophages. However, the combination of BMS-986351 and rituximab markedly increased the proportion of phagocytic macrophages in a dose-dependent manner. These results were replicated in OCI-Ly3 cells cocultured with macrophages derived from four different donors (Supplementary Fig. S4). These findings indicate that inhibition of the CD47-SIRPα antiphagocytic axis and activation of prophagocytic signaling with rituximab have an enhanced antitumor effect in DLBCL cell lines compared with rituximab alone.

### Species Cross-reactivity, Pharmacokinetics, and Safety

**FIGURE 5:**
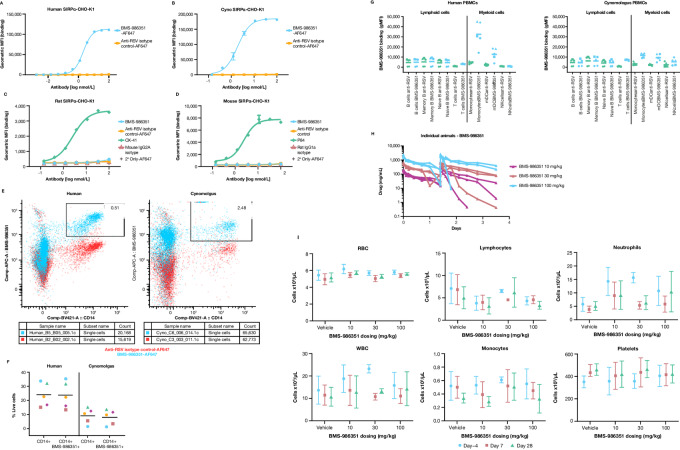
Cross-reactivity of BMS-986351 in human (**A**), cynomolgus monkey (**B**), rat (**C**), and mouse (**D**). **E,** Immunophenotyping by multiparameter flow cytometry in human and cynomolgus monkeys shows that most CD14-positive cells (monocytes) also bind BMS-986351. **F,** Proportion of live cells expressing CD14 that bind BMS-986351 in humans and cynomolgus monkeys. **G,** Binding of BMS-986351 in specific lymphoid and myeloid cell subsets. Pharmacokinetic profiles (day 1 to 15; **H**) and hematology assessment (days 4, 7, and 28; **I**) of BMS-986351 dosed in cynomolgus monkeys (*n* = 3) at 10, 30, and 100 mg/kg. MFI, mean fluorescence intensity.

The cross-reactivity of BMS-986351 with human, macaque, rat, and mouse SIRPα was assessed *in vitro* (Fig. 5A–D). Assays were performed in CHO-K1 cell lines overexpressing each species’ SIRPα, and cross-reactivity was measured by geometric mean fluorescence intensity. Results indicated cross-reactivity of BMS-986351 with humans and macaque SIRPα but not rodent SIRPα.

Immunophenotyping of samples from human and cynomolgus monkeys was performed using multiparameter flow cytometry. PBMCs were stained with BMS-986351 and various lineage-specific markers (Fig. 5E). Almost all the CD14-positive (monocyte specific) cells were costained for BMS-986351 in human and cynomolgus PBMCs, although the proportion of PBMCs that were CD14-positive was higher in humans than monkeys (Fig. 5F). This demonstrates that BMS-986351 was capable of binding almost all monocytes in the samples. A more detailed comparison of BMS-986351 binding in immune cell subsets in humans showed that binding predominantly occurred in cells of myeloid origin, including monocytes, and to a lesser extent in myeloid dendritic cells, while no binding was observed in natural killer (NK) cells (Fig. 5G). Among cells of lymphoid origin, no binding was observed in B cells, and minor activity was detected in CD3^+^ T cells, potentially attributable to SIRPγ expression in these cells. A similar pattern across immune cell subtypes was shown in cynomolgus monkeys. Further *in vitro* analyses indicated that BMS-986351 alone did not mediate antibody-dependent cell-mediated cytotoxicity by NK cells, nor did it induce autologous ADCP (Supplementary Fig. S5).

### BMS-986351 Pharmacokinetics and Hematologic Effects *In Vivo*

The pharmacokinetics of BMS-986351 after two doses (day 1 and day 15) in cynomolgus monkeys is summarized in Fig. 5H and Table 1. Maximum concentration increased by dose level (10, 30, or 100 mg/kg), and the half-life ranged from 163 to 227 hours across individual animals. Clearance rates ranged from 7.77 to 15.48 mL/day/kg. Assessment of hematologic parameters over 28 days indicated that BMS-986351 did not deplete cell counts, including the number of WBCs, monocytes, lymphocytes, and RBCs (Fig. 5I).

**TABLE 1:** Pharmacokinetics of BMS-986351 in cynomolgus monkeys (*n* = 3) after dosing on day 1 and day 15 (values shown are means of repeated measurements)

Study day	Dose (mg/kg)	T_max_ (h)	C_max_ (µg/mL)	Partial AUC_0–168hr_ (µg*h/mL)	T_1/2_ (h)	CL (mL/day/kg)	Vdss (mL/kg)
1	10	1.50	183	13,127	227	7.77	99.80
	30	0.25	632	45,159	185	8.59	80.47
	100	1.50	1,984	136,669	217	8.37	91.93
15	10	1.50	183	13,127	227	7.77	99.80
	30	1.50	664	52,434	163	10.50	61.70
	100	1.50	2,276	184,409	187	15.48	61.70

Abbreviations: AUC_0–168_, area under the serum concentration–time curve from 0 to 168 hours; C_max_, peak serum concentration; CL, total body clearance; h, hour; T_1/2_, half-life; T_max_, time at peak serum concentration; Vdss, volume of distribution.

## Discussion

Although many promising cancer immunotherapies target the adaptive immune system, their efficacy is limited to specific cancer types, and cancer cells may develop resistance to these treatments over time (44). Innate immunity also plays a significant role in cancer biology via direct and indirect mechanisms, such as the direct destruction of cancerous cells, detection of damage-associated molecular patterns by pattern recognition receptors, and cytokine- and chemokine-associated stimulation of proinflammatory responses in the tumor microenvironment (44, 45). Recent studies suggested that anti-CD47 treatments can facilitate an adaptive immune response via CD8^+^ T cells (46). Novel anticancer therapies can aid in tumor clearance by enhancing the natural ability of innate immune cells to target and eradicate cellular malignancies.

One such therapeutic strategy involves increasing the phagocytotic capabilities of tumor-associated macrophages by modulating macrophage–tumor cell surface signaling via the CD47-SIRPα axis. Numerous compounds targeting CD47 have been investigated as potential anticancer therapies (21–25, 47, 48). However, the ubiquitous expression pattern of CD47 necessitates the use of higher therapeutic doses to overcome potential antigen sink effects (3).

Although the development of anti-SIRPα strategies was initially limited by a lack of mouse cross-reactive agents and an extensive number of SIRPα polymorphisms (12, 49, 50), several anti-SIRPα approaches have been developed recently (11, 20, 49), including SIRPα-Fc fusion proteins currently being investigated in clinical trials (25, 51). The more restricted expression pattern of SIRPα may limit drug-related toxicities and, compared with CD47, lower doses may be expected to overcome target-mediated drug disposition (52). In this study, we report the discovery of BMS-986351, an anti-SIRPα IgG1 mAb with a lysine to alanine substitution that reduces its ability to activate complement while maintaining FcγR binding activity. Reducing complement activation removed any potential for autologous complement depletion/fixation of non-tumor cell populations, including monocytes, neutrophils, and other immune lineages expressing SIRPα. BMS-986351 potently blocks SIRPα-CD47 binding and has antitumor activity *in vitro*, and its effects are potentiated when combined with well-characterized and widely used therapeutic antibodies such as rituximab and cetuximab. Compared with SIRPα-Fc fusion proteins, an anti-SIRPα antibody, such as BMS-986351, provides a higher-affinity blocking capability to CD47, has single-agent activity, and has shown minimal impact on hematologic parameters, including thrombocytopenia (25, 51).

Extensive characterization of BMS-986351 provided insight into its biological effects. Cocrystallization studies confirmed that the antibody engages SIRPα in a region overlapping the binding site for CD47, providing a mechanism by which BMS-986351 prevents the SIRPα–CD47 interaction. Analysis of the 1kG dataset identified the six most prevalent SIRPα haplotypes, and BMS-986351 demonstrated broad binding coverage. To our knowledge, this is the first SIRPα antibody study to include haplotype identification.

Antibody characterization analyses revealed that BMS-986351 exhibited high binding affinity for SIRPγ in addition to SIRPα. However, immunophenotyping data across human and cynomolgus PBMCs demonstrated that BMS-986351 binding to T cells was significantly lower than to monocytes. Although SIRPγ binding has been described to affect transmigration and possibly T-cell activity during chronic stimulation (29, 53), it is currently unknown whether BMS-986351 binding to T cells will positively or negatively impact their activation and function in the clinical setting.

Target assessment via RNA sequencing and IHC confirmed the presence of SIRPα in bulk tumor samples from various solid and hematologic malignancies, and SIRPα expression correlated with the presence of macrophages. Targeting SIRPα with BMS-986351 led to potent inhibition of SIRPα-CD47 binding and increased phagocytic activity in *in vitro* models of colorectal cancer, SCCHN, and DLBCL cell lines. Combining BMS-986351 with cetuximab in colorectal cancer and SCCHN or with rituximab in DLBCL markedly increased macrophage phagocytosis, demonstrating that inhibition of the antiphagocytotic activity by BMS-986351 enhances prophagocytic signals from the opsonizing antibodies in these tumor types and may allow for specific tumor target-mediated phagocytosis. In our experiments, we did not find that the sequencing of BMS-986531 and cetuximab treatments impacted the results. Importantly, the efficacy of BMS-986351 and cetuximab in colorectal cancer cell lines with *KRAS* mutations represents a potential treatment opportunity for cetuximab-resistant tumors, given that the mechanism of action of BMS-986351 may be independent of EGFR pathway activity. Similar synergistic effects between agents blocking CD47 and opsonizing antibodies have been reported (42, 54).

Our immunophenotyping studies confirmed that BMS-986351 preferentially binds to monocytes. This was supported by our assessment of hematologic parameters in cynomolgus monkeys, which showed that intravenous delivery of BMS-986351 at therapeutic doses was safe, with no evidence of WBC, monocyte, lymphocyte, or RBC depletion over 28 days of exposure to BMS-986351. This differs from antibody treatments targeting CD47, which can cause anemia due to the expression of CD47 on RBCs (46).

In summary, we have characterized a novel, high-affinity fully human anti-SIRPα antibody that blocks SIRPα–CD47 interactions and promotes macrophage-mediated phagocytosis of tumor cells. BMS-986351 demonstrated increased phagocytic activity in models of solid tumors and hematologic malignancies, and the effects were enhanced when combined with cetuximab or rituximab. A phase I dose-escalation study is underway to evaluate BMS-986351 alone or in combination with other therapeutic antibodies in patients with advanced cancer (NCT03783403). Further research is needed to better characterize the impact of SIRPα inhibition in the tumor microenvironment and the potential interactions between BMS-986351 and other therapies, including immune checkpoint inhibitors.

## Supplementary Material

Supplementary Methods Preparation of BMS-986351

Supplementary Table S1 Abundance of the most prevalent CD47–SIRPα binding interface haplotypes

Supplementary Table S2 Binding affinity of SIRPα, SIRPβ, and SIRPγ, as determined by surface plasmon resonance

Supplementary Table S3 Inhibition of CD47–SIRPα binding by serially-diluted BMS-986351, as determined by surface plasmon resonance

Supplementary Table S4 Summary of FcγR binding EC50 determination of BMS-986351

Supplementary Table S5 Binding coverage of BMS-986351 across the six major SIRPα haplotypes

Supplementary Table S6 Pearson correlation coefficient (cc) of SIRPA expression vs each macrophage-related gene in each of the separate TCGA cohorts, sorted high to low based on the mean of the correlation coefficients

Supplementary Table S7 Incidences of CD163-positive macrophages and CD66b-positive neutrophil infiltrates in CRC, SCCHN, and DLBCL (A). Summary incidences of H-score values for SIRPα and EGFR in SCCHN (B) and DLBCL (C).

Supplementary Figure S1 CD47 gene expression across all of TCGA, shown with a boxplot for each indication (A), and a scatterplot showing the expression of SIRPA compared to the average macrophage gene expression (mean of CD163, CSF1R, CD14 and CD68 gene expression) in TCGA cohorts (B).

Supplementary Figure S2 Baseline CD47 expression by flow cytometry in colorectal cancer cell lines (A) and in DLBCL cell lines (B).

Supplementary Figure S3 Rituximab dose selection based on macrophage phagocytosis in CD20-positive NHL cell lines. Data showed that a rituximab dose of 0.1 nM induced maximal phagocytosis in OCI-Ly3 cells.

Supplementary Figure S4 Enhanced phagocytosis in OCI-Ly3 cells is achieved when BMS-986351 is combined with rituximab.

Supplementary Figure S5 Lack of BMS-986351 effect on NK-mediated ADCC of tumors cells (A), autologous normal monocytes and unactivated CD4 cells (B), and autologous ADCP activity on monocytes and T cells (C).
