# Supplementary material for: Discovery and Preclinical Activity of BMS-986351, an Antibody to SIRPα That Enhances Macrophage-mediated Tumor Phagocytosis When Combined with Opsonizing Antibodies
Source: Cancer Res Commun. 2024 Feb 22;4(2):505–15. doi: 10.1158/2767-9764.CRC-23-0634 (PMC10883291; doi:10.1158/2767-9764.CRC-23-0634)
Supplement: Supplementary Methods — Preparation of BMS-986351 [file crc-23-0634-s01.pdf]

SUPPLEMENTARY METHODS

Preparation of BMS-986351

Research grade BMS-986351 was manufactured at laboratory scale in 3L shake-flasks for in vitro characterization studies. Transient transfection of ExpiCHO cells using a Life Technologies (ThermoFisher Scientific) standard protocol, with a 1:1 ratio of light chain versus heavy chain DNA used for the DNA mixture at 0.5 mg/L of culture during the transfection. The VL and VH sequences are given below. Cells were seeded at $6x10^6$ cells/mL in a 3L shake flask with 1L working volume at 37°C plus 5% CO_2. On Day 1 post-transfection, standard enhancers 1 and 2 were added. Cell viability and titer were monitored every day and supernatant was harvested on Day 8 post-transfection, with viability monitoring and Octet RED (ForteBio, Fremont, CA) equipped with Protein A sensor was used for titer analysis. Cells and supernatant were harvested using GE Lifesciences depth filtration and sterilization columns. ULTA Prime GF 5 µm capsules were used for depth filtration followed by ULTA Pure HC 0.6/0.2 µm sterilization capsules (both from Cytiva, Marlborough, MA). Purification of the materials produced by BMS included Protein A affinity chromatography and low pH virus inactivation, followed by IEX interaction (Capto Adhere and Capto SP ImpRes; Cytiva, Marlborough, MA) chromatography steps. The purified antibody was bulk-formulated by buffer exchange into a suitable buffer and filtered through a 0.2 μm filter prior to storage.

The VL and VH sequences of BMS-985351 are below.

VL:

DIQMTQSPSSVSASVGDRVTITCRASQGISSWLAWYQQKPGKAPKLLIYAASNLQS
GVPSRFSGSGSGTDFTLTISSLQPEDFATYYCQQGASFPITFGGGTKVEIK

VH:

QVQLVQSGAEVKKPGASVKVSCKASGYTFRGYGISWVRQAPGQGLEWMGWISAY
GGETNYAQKLQGRVTMTTDTSTSTAYMELRSLRSDDTAVYYCAREAGSSWYDFD
LWGRGTLVTVSS
