# Supplementary material for: Discovery and Preclinical Activity of BMS-986351, an Antibody to SIRPα That Enhances Macrophage-mediated Tumor Phagocytosis When Combined with Opsonizing Antibodies
Source: Cancer Res Commun. 2024 Feb 22;4(2):505–15. doi: 10.1158/2767-9764.CRC-23-0634 (PMC10883291; doi:10.1158/2767-9764.CRC-23-0634)
Supplement: Supplementary Table S1 — Abundance of the most prevalent CD47–SIRPα binding interface haplotypes [file crc-23-0634-s02.pdf]

Supplementary Table S1. Abundance of the most prevalent CD47–SIRPα binding interface haplotypes.

Allele 1	Allele 2	Abundance	Haplotype Variants	Allele 1 Sequence	Allele 2 Sequence
HG01461.2	HG01461.2	735	1	RELIYNQKEGHFPRVTTVSDLTKRNNMDFSI	-
NA20127.2	NA20127.2	171	2	RELIYNQKEGHFPRVTTVSESTKRENMDFSI	-
HG01461.2	NA20127.2	116	1, 2	RELIYNQKEGHFPRVTTVSDLTKRNNMDFSI	RELIYNQKEGHFPRVTTVSESTKRENMDFSI
HG01461.2	NA19749.2	21	1, 3	RELIYNQKEGHFPRVTTVSDLTKRNNMDFSI	RELIYNQKEGHFPRVTTVSDLTKRENMDFSI
HG00159.2	NA20127.2	8	4, 2	RELIYNQKEGHFPRVTTVSESTKRKNMDFSI	RELIYNQKEGHFPRVTTVSESTKRENMDFSI
HG01461.2	NA18516.2	7	1, 5	RELIYNQKEGHFPRVTTVSDLTKRNNMDFSI	RELIYNQKEGHFPRVTTVSEPTKRNNMDFSI
HG01461.2	HG01242.2	7	1, 6	RELIYNQKEGHFPRVTTVSDLTKRNNMDFSI	RELIYNQKEGHFPRVTTVSELTKRENMDFSI

SIRPα = signal regulatory protein-α.
