# Supplementary material for: Discovery and Preclinical Activity of BMS-986351, an Antibody to SIRPα That Enhances Macrophage-mediated Tumor Phagocytosis When Combined with Opsonizing Antibodies
Source: Cancer Res Commun. 2024 Feb 22;4(2):505–15. doi: 10.1158/2767-9764.CRC-23-0634 (PMC10883291; doi:10.1158/2767-9764.CRC-23-0634)
Supplement: Supplementary Table S2 — Binding affinity of SIRPα, SIRPβ, and SIRPγ, as determined by surface plasmon resonance [file crc-23-0634-s03.pdf]

Supplementary Table S2. Binding affinity of SIRPα, SIRPβ, and SIRPγ, as determined by surface plasmon resonance.

Sample	k_a ($M^{-1}s^{-1}$)	k_d (s^{-1})	K_D (nM)	R_{max} (RU)	Chi^2 (RU^2)	Ligand (3.33 nM)	Model
SIRPα	4.84E+06	2.62E-04	0.0541	76.6	3.54	BMS-986351	1:1 Binding
SIRPβ	2.99E+06	6.13E-04	0.205	66.6	1.68	BMS-986351	1:1 Binding
SIRPγ	7.08E+06	3.55E-04	0.0502	72.1	2.8	BMS-986351	1:1 Binding

k_a = association constant, k_d = disassociation constant, K_D = equilibrium dissociation constant, R_{max} = maximum intrinsic rate of increase, SIRP = signal regulatory protein.
