# Supplementary material for: Discovery and Preclinical Activity of BMS-986351, an Antibody to SIRPα That Enhances Macrophage-mediated Tumor Phagocytosis When Combined with Opsonizing Antibodies
Source: Cancer Res Commun. 2024 Feb 22;4(2):505–15. doi: 10.1158/2767-9764.CRC-23-0634 (PMC10883291; doi:10.1158/2767-9764.CRC-23-0634)
Supplement: Supplementary Table S3 — Inhibition of CD47–SIRPα binding by serially-diluted BMS-986351, as determined by surface plasmon resonance [file crc-23-0634-s04.pdf]

Supplementary Table S3. Inhibition of CD47–SIRPα binding by serially-diluted BMS-986351, as determined by surface plasmon resonance.

BMS-986351 (nM)	Response (RU)	Calculated equilibrium concentration, B_{eq} (nM)	CD47 binding (%)	Inhibition (%)
0	307.6	18.56	100	0
0.4	298.1	17.55	96.91	3.09
1.2	275.7	15.36	89.63	10.37
3.7	239.1	12.23	77.73	22.27
11.11	167	7.306	54.29	45.71
33.33	25	0.8135	8.13	91.87
100	7.9	0.2523	2.57	97.43

SIRPα = signal regulatory protein-α.
