# Supplementary material for: Discovery and Preclinical Activity of BMS-986351, an Antibody to SIRPα That Enhances Macrophage-mediated Tumor Phagocytosis When Combined with Opsonizing Antibodies
Source: Cancer Res Commun. 2024 Feb 22;4(2):505–15. doi: 10.1158/2767-9764.CRC-23-0634 (PMC10883291; doi:10.1158/2767-9764.CRC-23-0634)
Supplement: Supplementary Table S4 — Summary of FcγR binding EC50 determination of BMS-986351 [file crc-23-0634-s05.pdf]

Supplementary Table S4. Summary of FcγR binding EC_{50} determination of BMS-986351.

FcyR isoform	Anti-RSV-IgG1 EC_{50} (nM)	BMS-986351 EC_{50} (nM)	Rituximab EC_{50} (nM)
FcγR1 (CD64)	1.2	0.8	0.8
FcγR2A (CD32aH131)	136.1	200.4	361.2
FcγR2A (CD32aR131)	90.2	188.2	194.2
FcγR2B	1200	2478	1325
FcγR3A (CD16aV158)	448.6	795.1	574.6
FcγR3A (CD16aF158)	311.3	1200	1270

EC_{50} = half-maximal effective concentration, FcγR = Fcγ receptor.
