# Supplementary material for: Discovery and Preclinical Activity of BMS-986351, an Antibody to SIRPα That Enhances Macrophage-mediated Tumor Phagocytosis When Combined with Opsonizing Antibodies
Source: Cancer Res Commun. 2024 Feb 22;4(2):505–15. doi: 10.1158/2767-9764.CRC-23-0634 (PMC10883291; doi:10.1158/2767-9764.CRC-23-0634)
Supplement: Supplementary Table S5 — Binding coverage of BMS-986351 across the six major SIRPα haplotypes [file crc-23-0634-s06.pdf]

Supplementary Table S5. Binding coverage of BMS-986351 across the six major SIRPα haplotypes.

**Haplotype**	**CD47–SIRPα Binding Interface**	**K_{on} ($M^{-1}s^{-1}$)**	**k_{off} (s^{-1})**	**K_D (M)**
v1 DLN	RELIYNQKEGHFPRVTTVSDLTKRNNMDFSI	1.49E+06	2.00E-04	1.34E-10
v2 ESE	RELIYNQKEGHFPRVTTVSESTKRENMDFSI	1.27E+06	5.59E-03	4.39E-09
v3 DLE	RELIYNQKEGHFPRVTTVSDLTKRENMDFSI	1.38E+06	2.00E-04	1.45E-10
v4 ESK	RELIYNQKEGHFPRVTTVSESTKRKNMDFSI	1.5E+06	2.28E-03	1.52E-09
v5 EPN	RELIYNQKEGHFPRVTTVSEPTKRNNMDFSI	1.73E+06	1.06E-03	6.11E-10
v6 ELE	RELIYNQKEGHFPRVTTVSELTKRENMDFSI	1.94E+-6	3.56E-04	1.83E-10

K_D = equilibrium dissociation constant, k_{on} = association rate constant, k_{off} = dissociation rate constant, SIRPα = signal regulatory protein-α.
