# Supplementary material for: Discovery and Preclinical Activity of BMS-986351, an Antibody to SIRPα That Enhances Macrophage-mediated Tumor Phagocytosis When Combined with Opsonizing Antibodies
Source: Cancer Res Commun. 2024 Feb 22;4(2):505–15. doi: 10.1158/2767-9764.CRC-23-0634 (PMC10883291; doi:10.1158/2767-9764.CRC-23-0634)
Supplement: Supplementary Table S6 — Pearson correlation coefficient (cc) of SIRPA expression vs each macrophage-related gene in each of the separate TCGA cohorts, sorted high to low based on the mean of the correlation coefficients [file crc-23-0634-s07.pdf]

Supplementary Table S6. Pearson correlation coefficient (cc) of SIRPA expression vs each macrophage-related gene in each of the separate TCGA cohorts, sorted high to low based on the mean of the correlation coefficients.

Gene	cc.HNSC	cc.COAD	cc.READ	cc.DLBC	Mean
CD68	0.376	0.558	0.599	0.778	0.578
CSF1R	0.379	0.656	0.564	0.674	0.568
CD14	0.332	0.601	0.586	0.596	0.529
MRC1	0.377	0.639	0.521	0.455	0.498
FCGR1A	0.164	0.605	0.530	0.629	0.482
CD163	0.289	0.629	0.548	0.451	0.479
C1QA	0.255	0.567	0.500	0.392	0.429
MARCO	0.110	0.575	0.423	0.472	0.395
ARG1	0.059	0.116	0.244	0.418	0.209

COAD = colon cancer, DLBC = diffuse large B-cell lymphoma, HNSC = head and neck cancer, READ = rectal cancer, SIRP = signal regulatory protein. TCGA = The Cancer Genome Atlas.
