# Supplementary material for: Discovery and Preclinical Activity of BMS-986351, an Antibody to SIRPα That Enhances Macrophage-mediated Tumor Phagocytosis When Combined with Opsonizing Antibodies
Source: Cancer Res Commun. 2024 Feb 22;4(2):505–15. doi: 10.1158/2767-9764.CRC-23-0634 (PMC10883291; doi:10.1158/2767-9764.CRC-23-0634)
Supplement: Supplementary Table S7 — Incidences of CD163-positive macrophages and CD66b-positive neutrophil infiltrates in CRC, SCCHN, and DLBCL (A). Summary incidences of H-score values for SIRPα and EGFR in SCCHN (B) and DLBCL (C). [file crc-23-0634-s08.pdf]

Supplementary Table S7. Incidences of CD163-positive macrophages and CD66b-positive neutrophil infiltrates in CRC, SCCHN, and DLBCL (**A**)**.** Summary incidences of H-score values for SIRPα and EGFR in SCCHN (**B**) and DLBCL (**C**). Grade 0 = none, Grade 1 = minimal, Grade 2 = mild, Grade 3 = moderate, Grade 4 = marked. Total H-score equals the product of the percent of tumor-positive cells and staining intensity (0 = none, 1 = weak, 2 = moderate, 3 = strong).

A

	**CRC (*n* = 97)**		**SCCHN (*n* = 80)**		**DLBCL (*n* = 75)**
**Cell Infiltrate**	**Macrophages (CD163)**	**Neutrophils (CD66b)**	**Macrophages (CD163)**	**Neutrophils (CD66b)**	**Macrophages (CD163)**
**Grade 0**	0 (0%)	21 (22%)	0 (0%)	9 (11%)	5 (7%)
**Grade 1**	28 (29%)	50 (52%)	14 (18%)	43 (55%)	10 (13%)
**Grade 2**	43 (44%)	13 (13%)	35 (43%)	17 (22%)	18 (24%)
**Grade 3**	18 (19%)	8 (8%)	20 (25%)	7 (9%)	23 (31%)
**Grade 4**	8 (8%)	5 (5%)	11 (14%)	2 (3%)	11 (15%)

B

IHC Marker	H-Score			
	**Negative**	**<100**	**100-200**	**>200**
**SIRPα**	12 (16%)	16 (21%)	39 (52%)	8 (11%)
**EGFR**	8 (11%)	24 (32%)	40 (53%)	5 (7%)

C

IHC Marker	H-Score			
	**Negative**	**<100**	**100-200**	**>200**
**SIRPα**	54 (72%)	3 (4%)	15 (20%)	3 (4%)

CRC = colorectal carcinoma, DLBCL = diffuse large B-cell lymphoma, EGFR = epidermal growth factor receptor, IHC = immunohistochemistry, SCCHN = squamous cell carcinoma head and neck, SIRPα = signal regulatory protein-α
