# Supplementary material for: Discovery and Preclinical Activity of BMS-986351, an Antibody to SIRPα That Enhances Macrophage-mediated Tumor Phagocytosis When Combined with Opsonizing Antibodies
Source: Cancer Res Commun. 2024 Feb 22;4(2):505–15. doi: 10.1158/2767-9764.CRC-23-0634 (PMC10883291; doi:10.1158/2767-9764.CRC-23-0634)

Supplementary Figure S1. CD47 gene expression across all of TCGA, shown with a boxplot for each indication (**A**), and a scatterplot showing the expression of SIRPA compared to the average macrophage gene expression (mean of CD163, CSF1R, CD14 and CD68 gene expression) in TCGA cohorts (**B**). TCGA, The Cancer Genome Atlas

A.

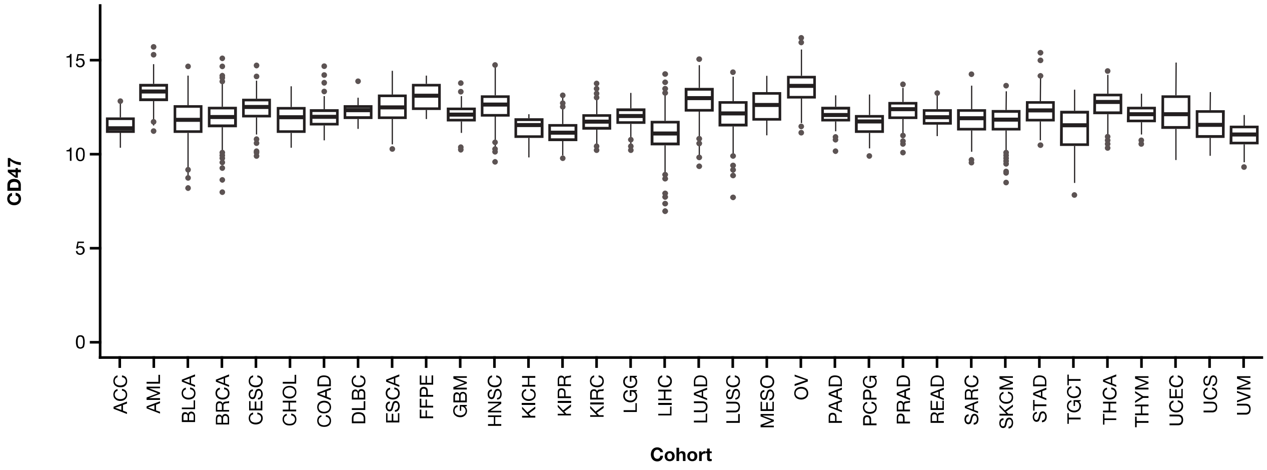

B.

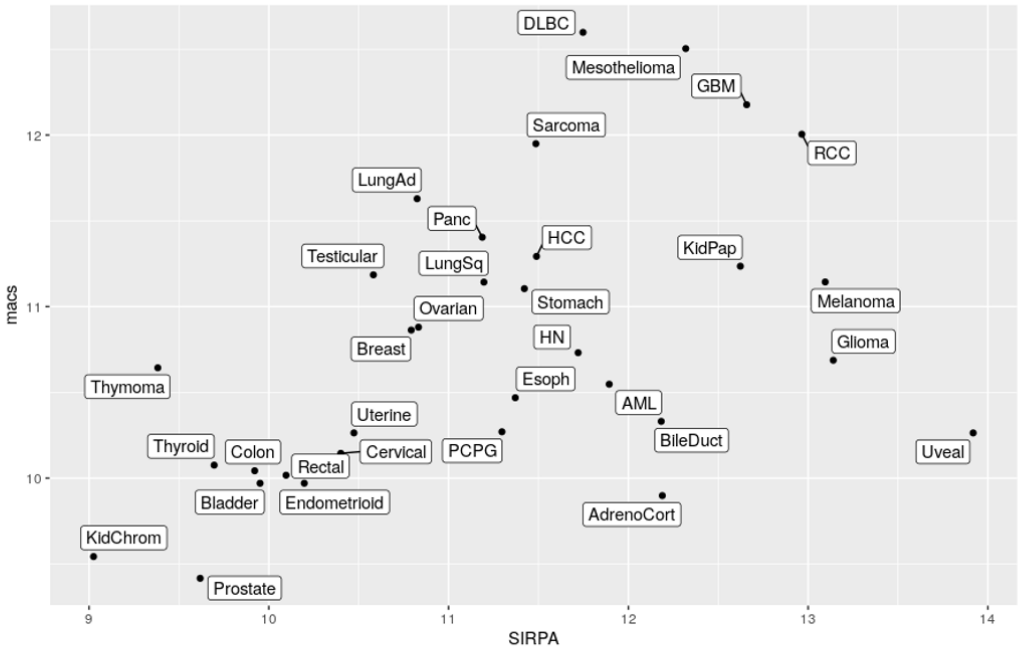

Supplement: Supplementary Figure S1 — CD47 gene expression across all of TCGA, shown with a boxplot for each indication (A), and a scatterplot showing the expression of SIRPA compared to the average macrophage gene expression (mean of CD163, CSF1R, CD14 and CD68 gene expression) in TCGA cohorts (B). [file crc-23-0634-s09.pdf]
