# Supplementary material for: Discovery and Preclinical Activity of BMS-986351, an Antibody to SIRPα That Enhances Macrophage-mediated Tumor Phagocytosis When Combined with Opsonizing Antibodies
Source: Cancer Res Commun. 2024 Feb 22;4(2):505–15. doi: 10.1158/2767-9764.CRC-23-0634 (PMC10883291; doi:10.1158/2767-9764.CRC-23-0634)

Supplementary Figure S2. Baseline CD47 expression by flow cytometry in colorectal cancer cell lines (**A**) and in DLBCL cell lines (**B**). DLBCL = diffuse large B-cell lymphoma.

A.

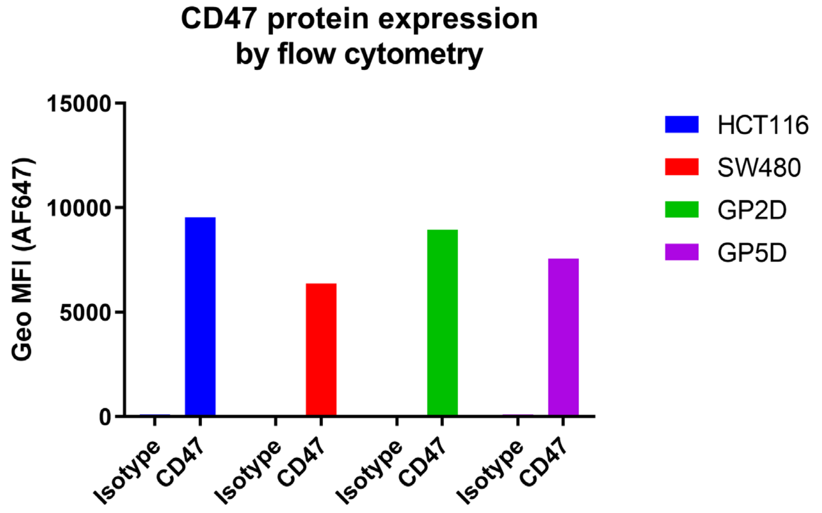

B.

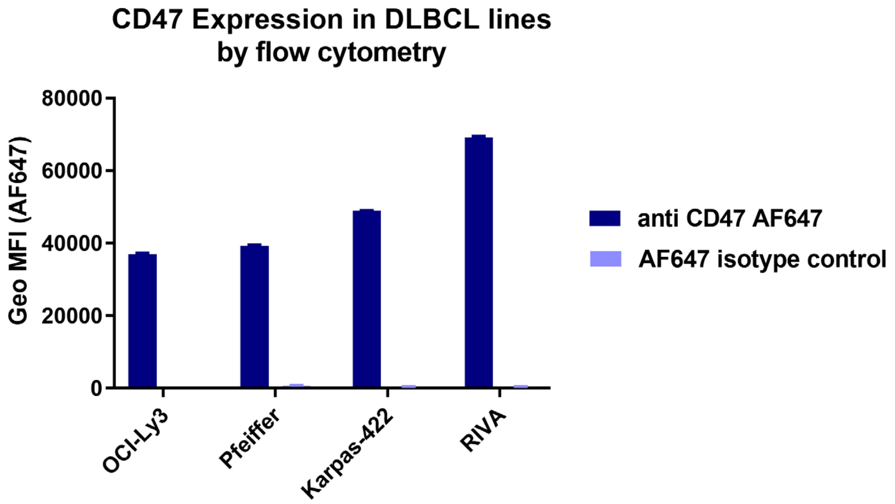

Supplement: Supplementary Figure S2 — Baseline CD47 expression by flow cytometry in colorectal cancer cell lines (A) and in DLBCL cell lines (B). [file crc-23-0634-s10.pdf]
