# Supplementary material for: Discovery and Preclinical Activity of BMS-986351, an Antibody to SIRPα That Enhances Macrophage-mediated Tumor Phagocytosis When Combined with Opsonizing Antibodies
Source: Cancer Res Commun. 2024 Feb 22;4(2):505–15. doi: 10.1158/2767-9764.CRC-23-0634 (PMC10883291; doi:10.1158/2767-9764.CRC-23-0634)

Supplementary Figure S3. Rituximab dose selection based on macrophage phagocytosis in CD20-positive NHL cell lines. Data showed that a rituximab dose of 0.1 nM induced maximal phagocytosis in OCI-Ly3 cells. NHL = non-Hodgkin lymphoma.

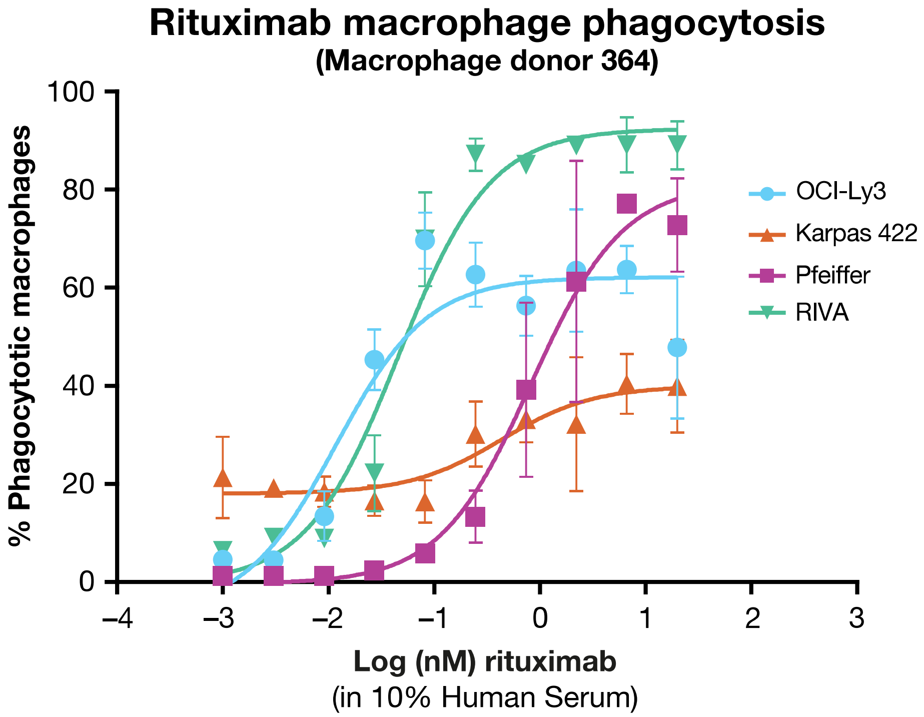

Supplement: Supplementary Figure S3 — Rituximab dose selection based on macrophage phagocytosis in CD20-positive NHL cell lines. Data showed that a rituximab dose of 0.1 nM induced maximal phagocytosis in OCI-Ly3 cells. [file crc-23-0634-s11.pdf]
