# Supplementary material for: Discovery and Preclinical Activity of BMS-986351, an Antibody to SIRPα That Enhances Macrophage-mediated Tumor Phagocytosis When Combined with Opsonizing Antibodies
Source: Cancer Res Commun. 2024 Feb 22;4(2):505–15. doi: 10.1158/2767-9764.CRC-23-0634 (PMC10883291; doi:10.1158/2767-9764.CRC-23-0634)
Supplement: Supplementary Figure S4 — Enhanced phagocytosis in OCI-Ly3 cells is achieved when BMS-986351 is combined with rituximab. [file crc-23-0634-s12.pdf]

Supplementary Figure S4. Enhanced phagocytosis in OCI-Ly3 cells is achieved when BMS-986351 is combined with rituximab. RSV = respiratory syncytial virus.

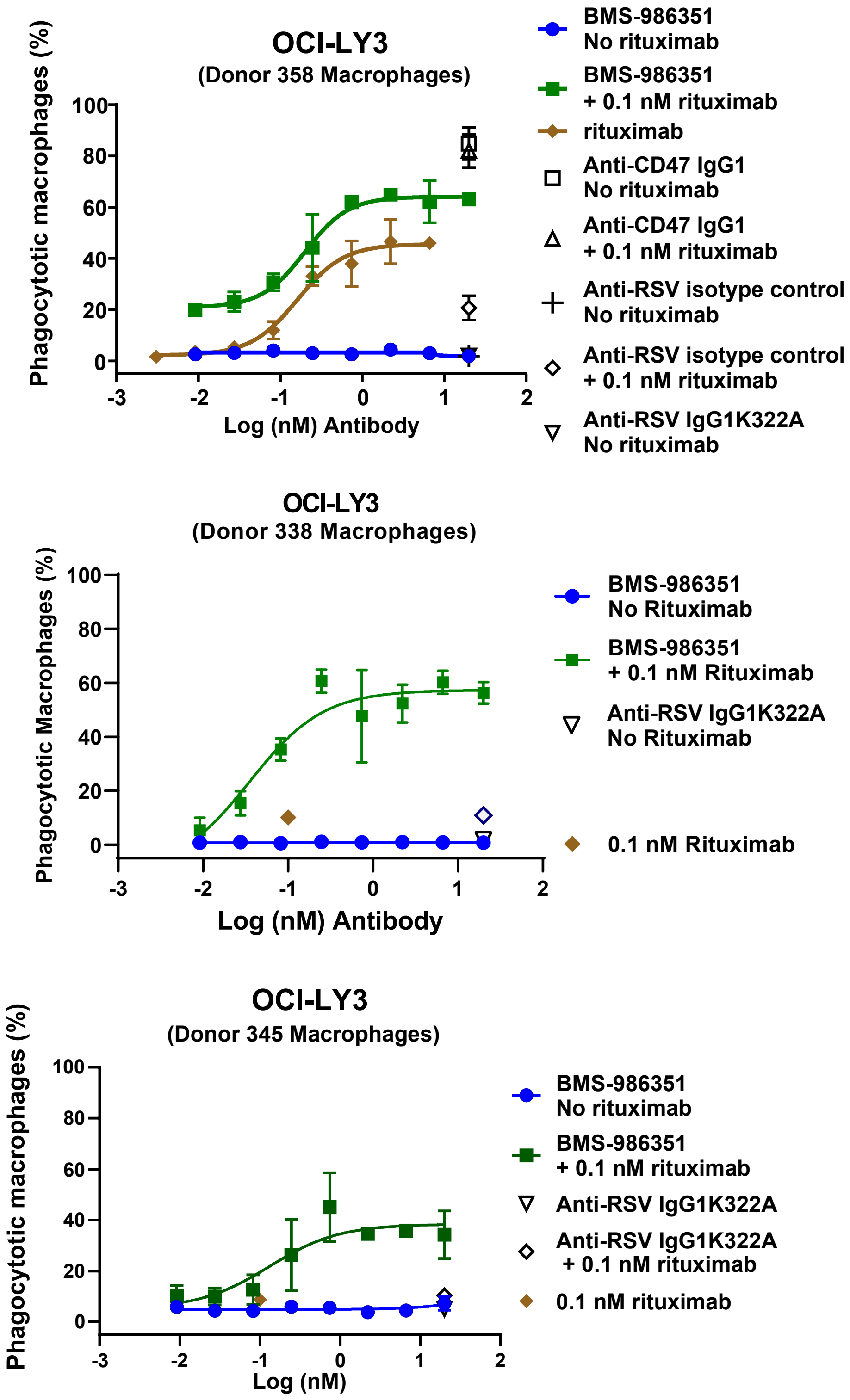

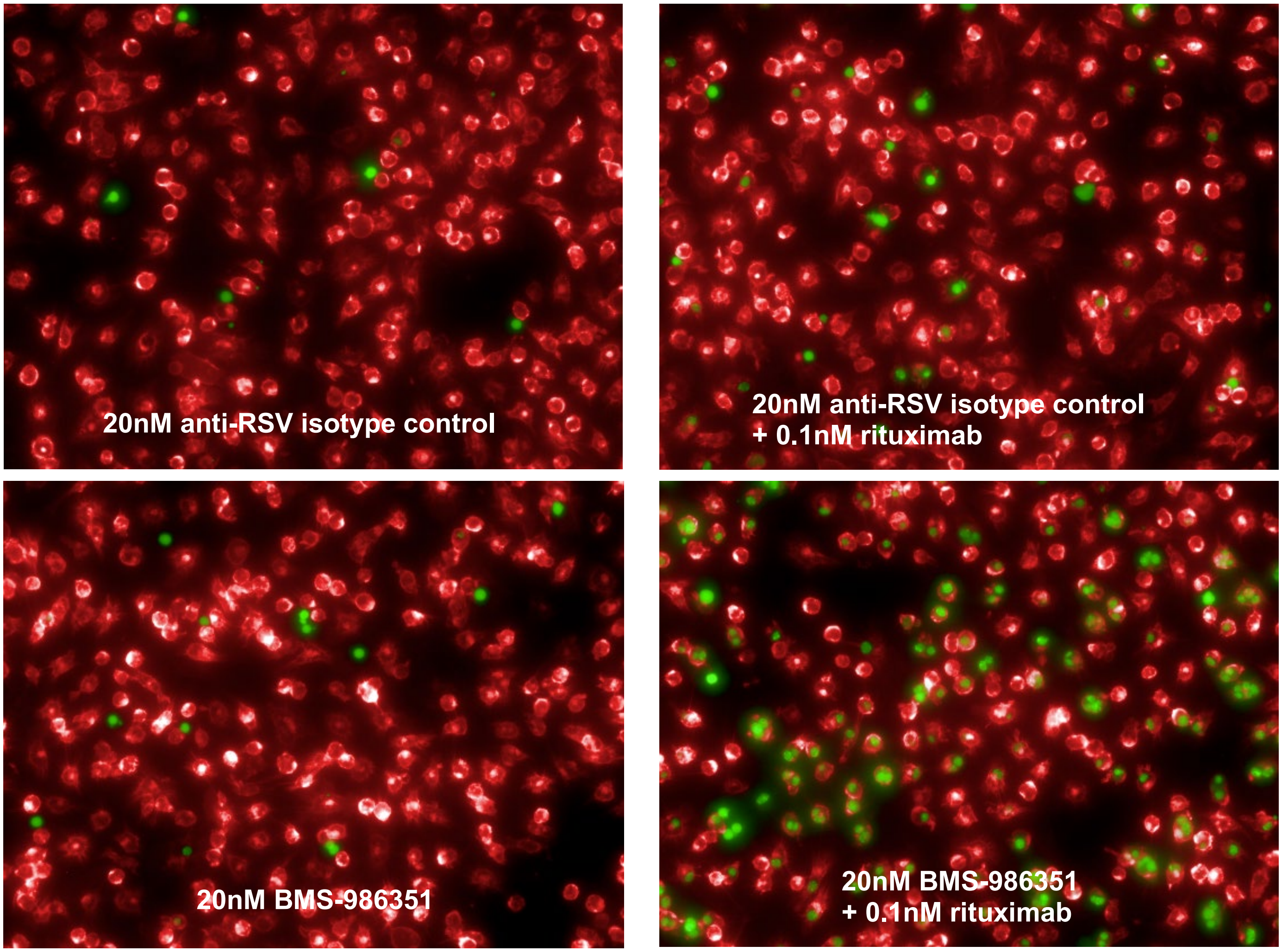
20nM anti-RSV isotype control
20nM anti-RSV isotype control
+ 0.1nM rituximab
20nM BMS-986351
20nM BMS-986351
+ 0.1nM rituximab
