# Supplementary material for: Discovery and Preclinical Activity of BMS-986351, an Antibody to SIRPα That Enhances Macrophage-mediated Tumor Phagocytosis When Combined with Opsonizing Antibodies
Source: Cancer Res Commun. 2024 Feb 22;4(2):505–15. doi: 10.1158/2767-9764.CRC-23-0634 (PMC10883291; doi:10.1158/2767-9764.CRC-23-0634)

Supplementary Figure S5. Lack of BMS-986351 effect on NK-mediated ADCC of tumors cells (**A**), autologous normal monocytes and unactivated CD4 cells (**B**), and autologous ADCP activity on monocytes and T cells (**C**). ADCC = antibody-dependent cell-mediated cytotoxicity, ADCP = antibody-dependent cellular phagocytosis, NK = natural killer.

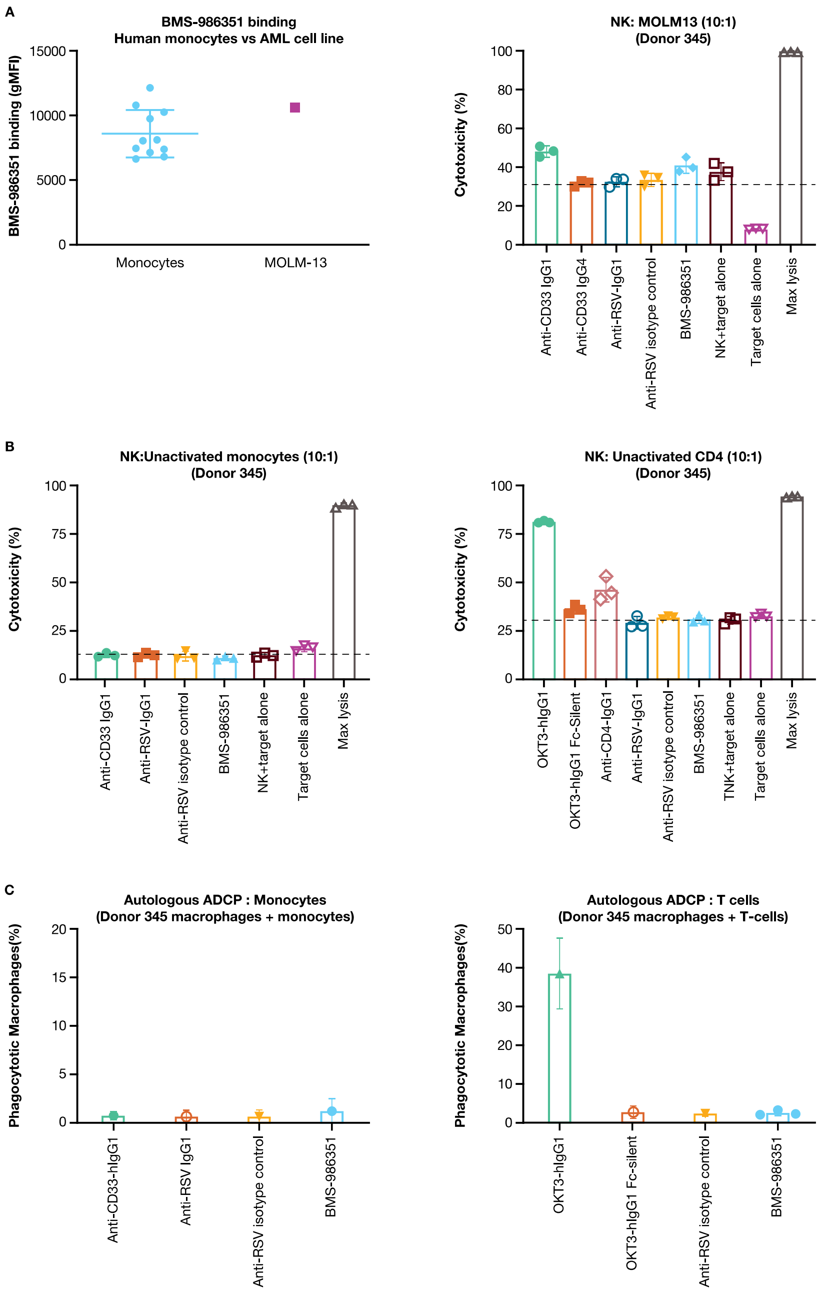

Supplement: Supplementary Figure S5 — Lack of BMS-986351 effect on NK-mediated ADCC of tumors cells (A), autologous normal monocytes and unactivated CD4 cells (B), and autologous ADCP activity on monocytes and T cells (C). [file crc-23-0634-s13.pdf]
